# Supplementary material for: The H3K9 methyltransferase SETDB1 maintains female identity in Drosophila germ cells
Source: Nat Commun. 2018 Oct 8;9:4155. doi: 10.1038/s41467-018-06697-x (PMC6175928; doi:10.1038/s41467-018-06697-x)
Supplement: Supplementary file 1 — Supplementary Information [file 41467_2018_6697_MOESM1_ESM.pdf]

Supplemental Information

The H3K9 methyltransferase SETDB1 maintains female identity in *Drosophila* germ cells

Anne E. Smolko, Laura Shapiro-Kulnane and Helen K. Salz

a **b**

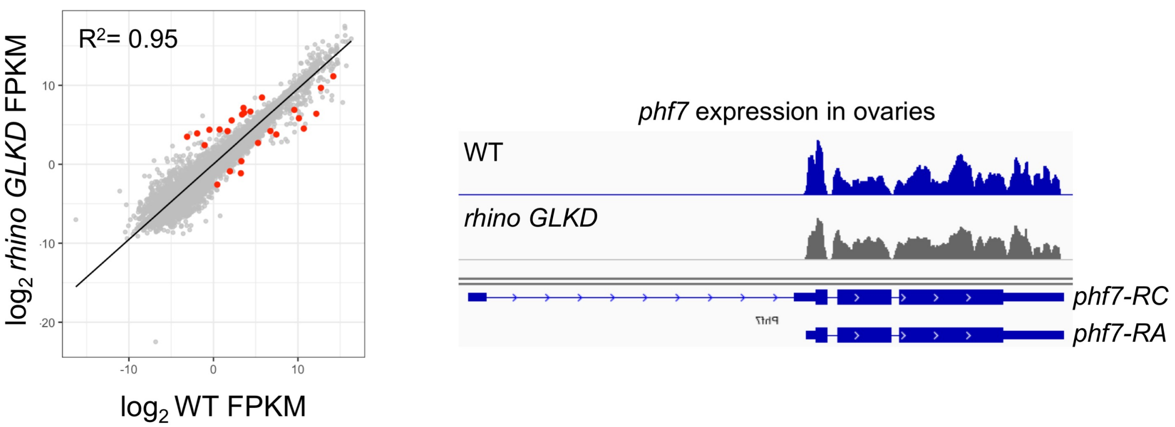

Supplementary Fig. 1 Loss of *rhino* in germ cells does not lead to global changes in gene expression. **a** Scatter plot comparing gene expression in *rhino* mutant ovaries (log_2, FPKM) relative to wild-type (WT). The 28 genes with at least a two-fold change in expression (FDR < 0.05) are highlighted in red. **b** *rhino* does not control sex-specific *phf7* transcription. Genome browser view of the *phf7* locus. Tracks show RNA-seq reads aligned to the *Drosophila* genome (UCSC dm6). All tracks are viewed at the same scale. The screen shot is reversed so that the 5' end of the gene is on the left. The two *phf7* transcripts, *phf7-RA* and *phf7-RC,* are indicated. *phf7-RA* is normally expressed in ovaries, and is expressed in both wild-type and *rhino* mutant ovaries. *phf7-RC* is normally only expressed in testis, and is not expressed in *rhino* mutant ovaries.

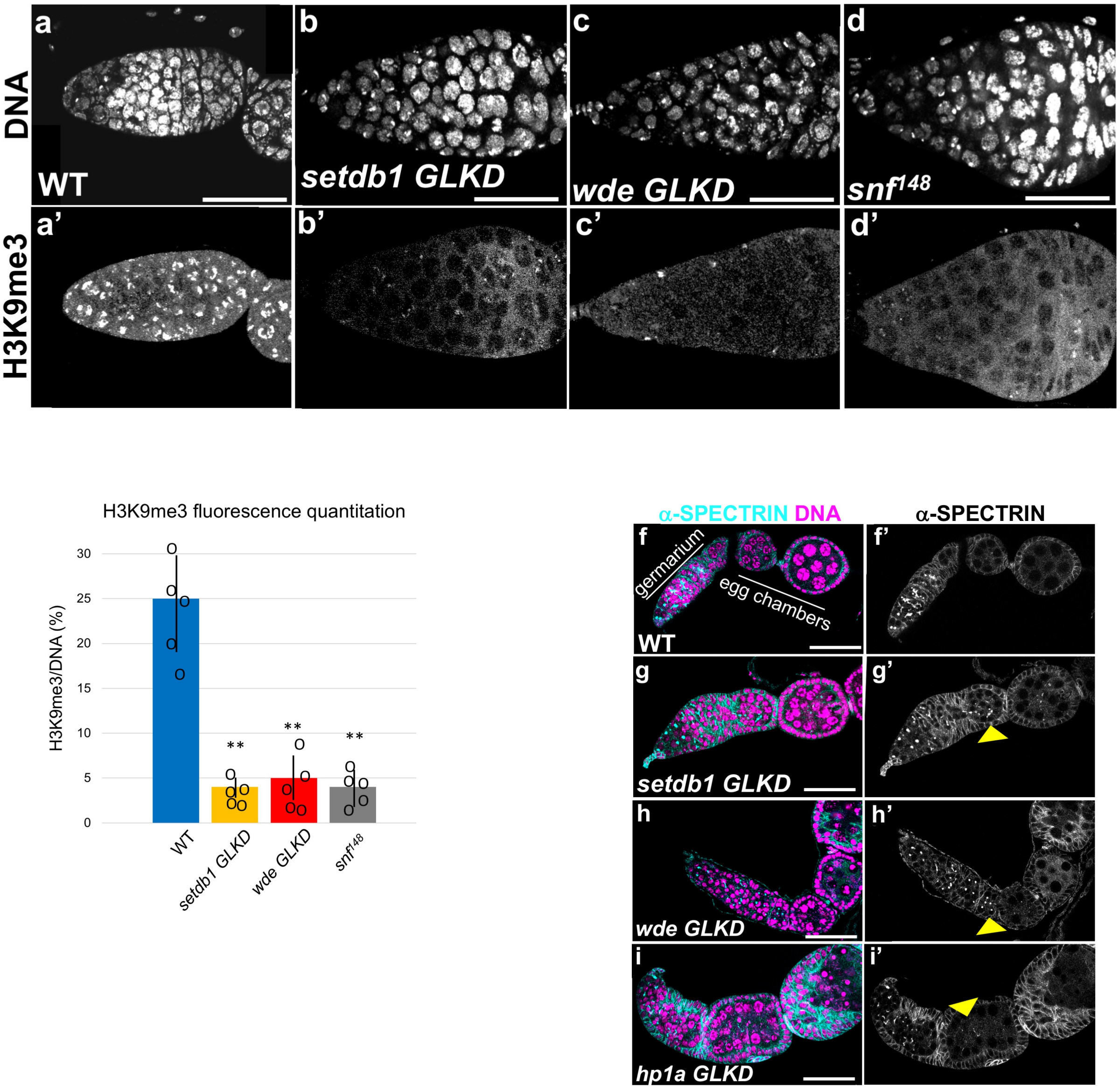

Supplementary Fig. 2 H3K9me3 pathway members are required for oogenesis. **a-d** H3K9me3 levels in WT and mutant ovaries. Representative confocal images of a wild-type (WT), *setdb1 GLKD, wde GLKD,* and *snf*^{148} germaria stained for DNA and H3K9me3. Scale bar, 25 μm. **e** Quantification of H3K9me3 intensity, normalized to DNA, in 5 wild-type and mutant germaria. Error bars indicate standard deviation. Significance of the difference between wild-type and mutant was determined by calculating p-values by Student's T-test from 5 replicates. **$p<0.01$. **f-i** Undifferentiated germ cells accumulate in *setdb1 GLKD, wde GLKD,* and *hp1a GLKD* mutant ovarioles. Representative confocal images of wild-type (WT) and mutant ovarioles stained for DNA (magenta) and α-spectrin (cyan, white in f'-i') to visualize spectrosomes, fusomes, and somatic cell membranes. Egg chambers in mutant ovarioles contain germ cells that retain spectrosomes (yellow arrow head).

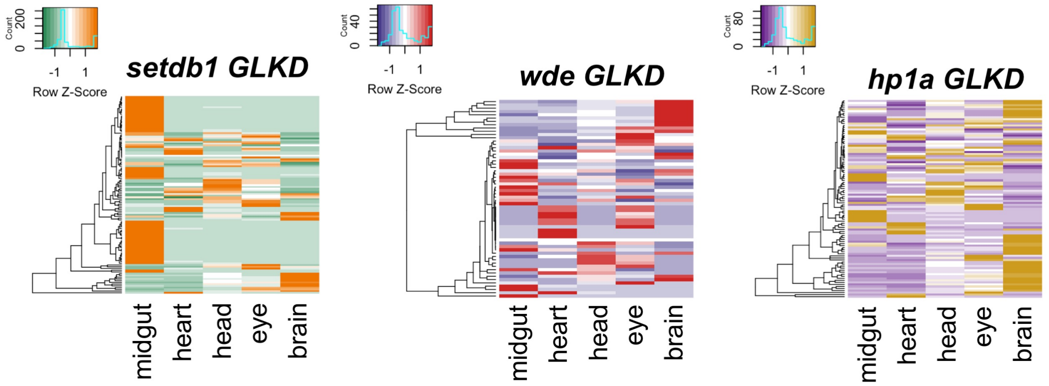

Supplementary Fig. 3 Aside from the testis, no predominant tissue-specific signature identified amongst the genes repressed by H3K9me3 SETDB1, WDE and HP1a. Hierarchical clustering of the tissue expression profiles of the ectopically expressed genes not normally expressed in testis genes. Gene expression per tissue (normalized to fly average) is shown as a z-score heatmap.

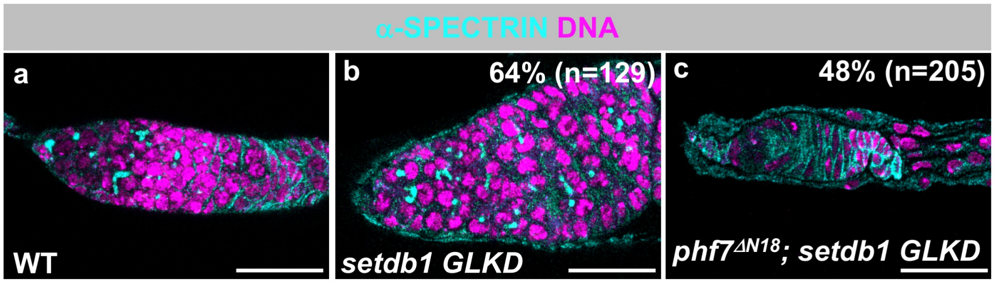

d

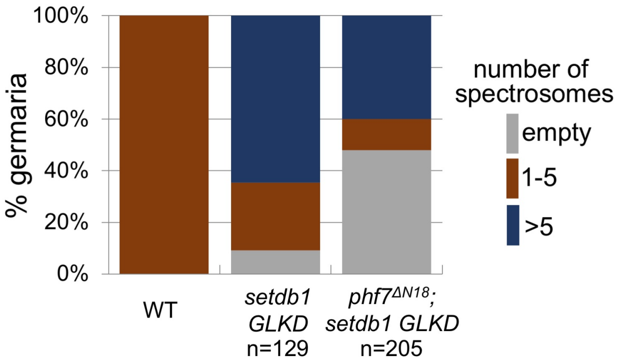

Supplementary Fig. 4 Ectopic *phf7* is required for *setdb1 GLKD* tumor growth. **a-c** Germaria from wild-type, *setdb1GLKD,* or double mutant *phf7*$^{\Delta 18}$/*phf7*$^{\Delta 18}$*; setdb1 GLKD* females stained for DNA (magenta) and α-spectrin (cyan) to visualize spectrosomes, fusomes, and somatic cell membranes. Scale bar, 25 μm. **d** Quantification of mutant germaria with 0, 1-5, and >5 round spectrosome-containing germ cells. The number of scored germaria (n) is indicated.

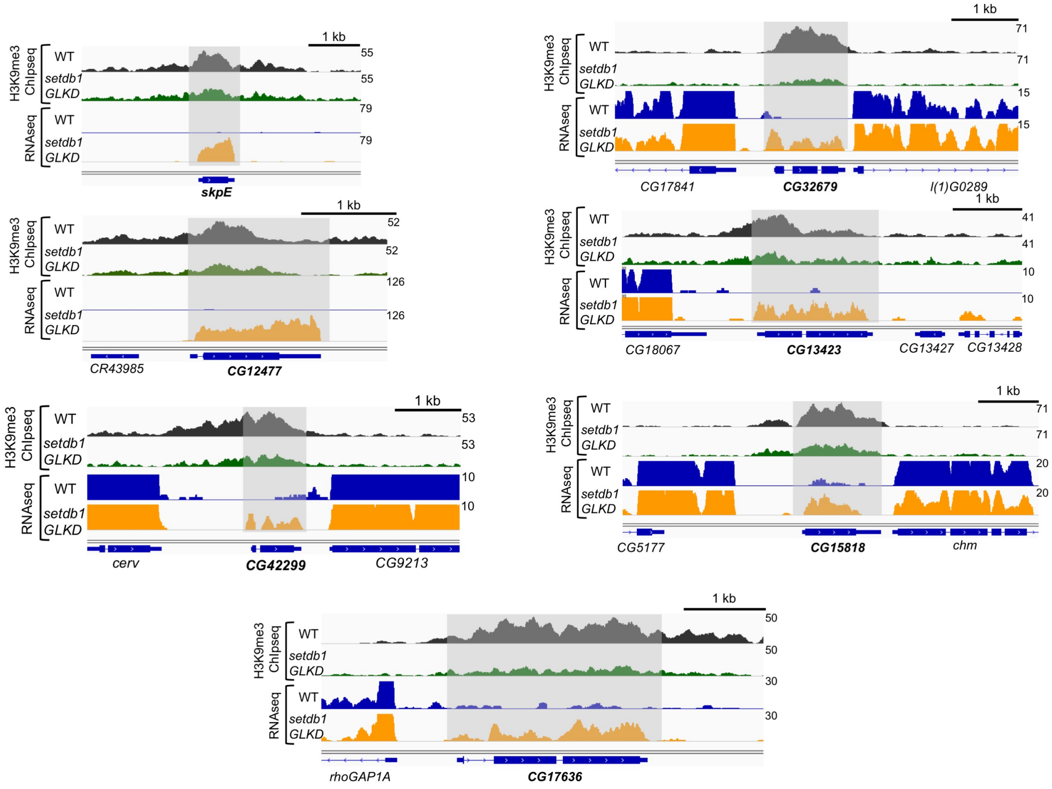

Supplementary Fig. 5 Examples of SETDB1/H3K9me3 regulated genes. Genome browser views of gene neighborhoods illustrate that the H3K9me3 islands present in WT ovaries does not spread to neighboring loci. In each example, shading highlights the gene whose ectopic expression in *setdb1* GLKD is correlated with a decreased H3K9me3 ChIP-seq peak.

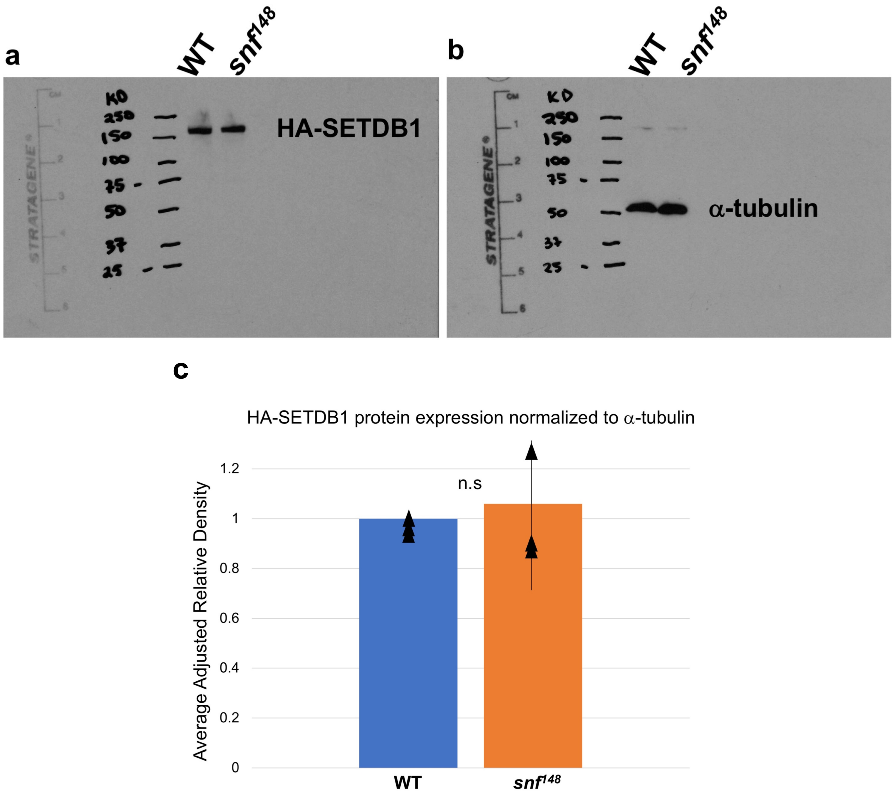

Supplementary Fig. 6 SETDB1 protein expression is not altered in *snf^{148}* mutants. **a-b** Full view of cropped Western Blots presented in Fig. 6e. Western blot of lysates made from ovaries dissected from wild-type and *snf^{148}* females carrying a copy an endogenously HA-tagged allele of *setdb1* probed with an antibody against the HA tag and the α-tubulin protein as a loading control. **c** Quantitation comparing wild-type (WT) and mutant levels of HA normalized to α-tubulin. Error bars indicate standard deviation (s.d). The significance of the differences between wild-type and mutant was determined by calculating p-values by Student's T-test from 3 replicates.

Supplementary Table 1

Location of SETDB1/H3K9me3 regulated genes

gene	Chromosome & sequence location	Cytogenetic map	Distance from nearest neighbor (Mb)
CG17636	X:124,370..126,714 [-]	1A	--
CG34434	X:5,614,554..5,616,370 [-]	5A8	5.5
Lim1	X:8,756,539..8,805,804 [-]	8A5-8B2	3.1
CG32679	X:10,366,402..10,367,472 [-]	9B12	1.6
Rab3-GEF	X:15,089,682..15,113,762 [+]	13A10-12	4.7
CG42299	X:15,574,947..15,575,683 [-]	13D	0.5
CR43299	X:15,662,289..15,665,707 [-]	13E	0.1
SkpE	X:19,823,368..19,824,080 [-]	18F	4.2
Phf7	X:20,160,355..20,165,830 [-]	19B3-19C1	0.3
CG32506	X:20,449,331..20,467,065 [+]	19D1	0.3
CG12061	X:23,036,066..23,047,678 [+]	20F4	2.6
CG15818	2L:7,410,909..7,412,229 [+]	27F3	--
CG15172	2L:19,022,417..19,023,646 [+]	37B9	11.6
CG13423	2R:20,536,578..20,538,264 [+]	57A5	--
CG10440	2R:21,560,246..21,576,035 [-]	57F1-2	1.0
MsR1	3L:2,322,693..2,345,799 [+]	62D6	--
CG12607	3L:4,446,573..4,448,072 [+]	64B6	2.1
CG10483	3L:5,917,905..5,921,316 [-]	64F5	1.5
CG12477	3L:18,721,893..18,723,272 [-]	75D7	12.8
CG42613	3R:18,929,196..18,978,088 [+]	91D5-91E1	--
CG31202	3R:29,928,701..29,930,386 [-]	99C7	10.9
