# Supplementary material for: The H3K9 methyltransferase SETDB1 maintains female identity in Drosophila germ cells
Source: Nat Commun. 2018 Oct 8;9:4155. doi: 10.1038/s41467-018-06697-x (PMC6175928; doi:10.1038/s41467-018-06697-x)
Supplement: Supplementary file 3 — Description of Additional Supplementary Files [file 41467_2018_6697_MOESM3_ESM.pdf]

Description of Additional Supplementary Files

File Name: Supplementary Data 1
Description: Genes upregulated at least 2-fold in setdb1 GLKD ovaries.

File Name: Supplementary Data 2
Description: Genes upregulated at least 2-fold in wde GLKD ovaries.

File Name: Supplementary Data 3
Description: Genes upregulated at least 2-fold in hp1a GLKD ovaries.

File Name: Supplementary Data 4
Description: Genes downregulated at least 2-fold in setdb1 GLKD ovaries.

File Name: Supplementary Data 5
Description: Genes downregulated at least 2-fold in wde GLKD ovaries.

File Name: Supplementary Data 6
Description: Genes downregulated at least 2-fold in hp1a GLKD ovaries.

File Name: Supplementary Data 7
Description: Many of the genes ectopically expressed in setdb1 GLKD ovaries are normally expressed in testis.

File Name: Supplementary Data 8
Description: Many of the genes ectopically expressed in wde GLKD ovaries are normally expressed in testis.

File Name: Supplementary Data 9
Description: Many of the genes ectopically expressed in hp1a GLKD ovaries are normally expressed in testis.
